# Intrapericardial migration of dislodged sternal struts as late complication of open pectus excavatum repairs

**DOI:** 10.1186/1749-8090-6-40

**Published:** 2011-03-30

**Authors:** Ruoyu Zhang, Christian Hagl, Dmitry Bobylev, Thomas Breymann, Jan D Schmitto, Axel Haverich, Marcus Krüger

**Affiliations:** 1 Division of Cardiac, Thoracic, Transplantation and Vascular Surgery, Hannover Medical School, Carl-Neuberg-Str. 1, 30625 Hannover, Germany

## Abstract

**Abstract:**

We present a case of sternal steel strut dislodgement and migration in a patient undergoing Ravitch repair for pectus excavatum (PE) 37 years ago. Broken struts perforated the right ventricle and right ventricular outflow tract (RVOT) and additionally migrated into the left upper lobar bronchus.

Dislodged sternal struts represent rare complications after surgical repair of patients suffering from pectus excavatum. Reviewing the literature, only five cases of intrapericardial migration of dislodged sternal struts or wires have been reported so far.

In our case, the first strut was removed from the airways through a left antero-lateral thoracotomy. Using cardiopulmonary bypass, a second strut was removed via ventriculotomy. These life-threatening sequelae underscore the importance of postoperative follow-up and early removal of osteosynthetic materials used in open PE repair. Accurate preoperative localization of migrated materials and availability of CPB support are crucial for successful surgical removal.

**Introduction:**

The migration of dislodged sternal steel struts or wires into the pericardium and cardiac cavities is a rare but life-threatening complication of open pectus excavatum (PE) repair [1]. Removal of these materials poses a challenge for cardiothoracic surgeons. Herein, the authors report a case of migration of dislodged steel struts through the right ventricle and right ventricular outflow tract (RVOT) into the left upper lobar bronchus in a patient who underwent Ravitch repair 37 years ago.

## **Case report**

A 53-year-old male with known persistent atrial fibrillation was admitted with progressive dyspnea and malaise, aggravated in sitting and stooping position. He had undergone surgical Ravitch repair for PE deformity 37 years ago.

**Figure 1:**
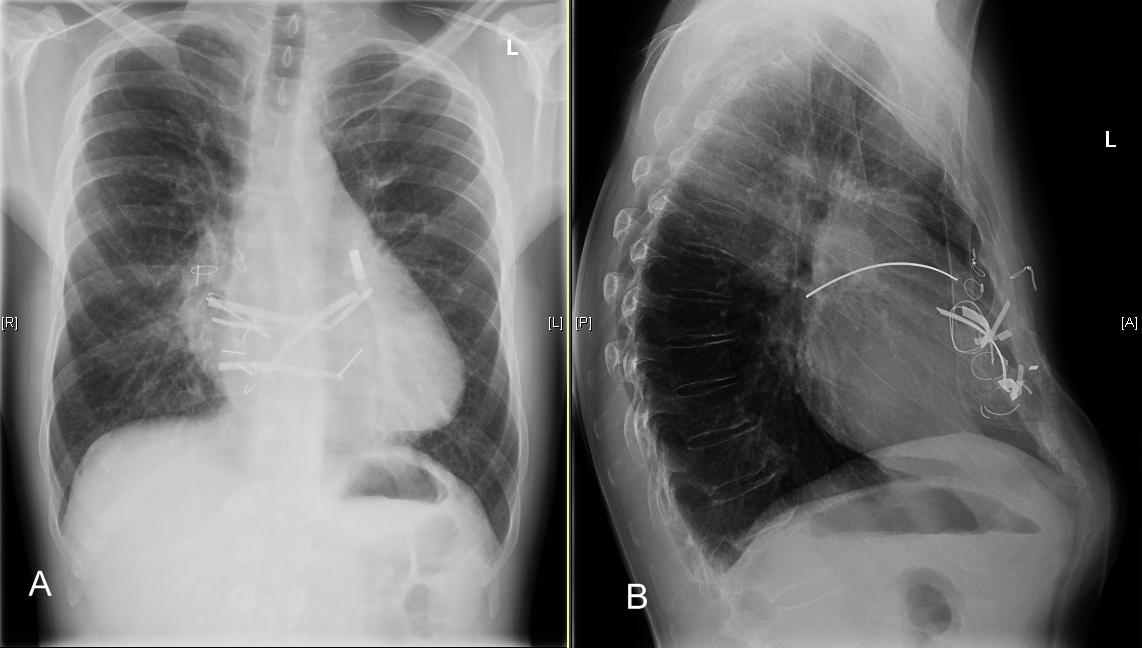
**The preoperative chest radiograph**. A: The chest radiograph demonstrated multiple dislodged steel struts and wires in a patient, who underwent Ravitch repair for pectus excavatum deformity 37 years ago. B: In the lateral view, several struts seemed to lie in the middle mediastinum.

**Figure 2:**
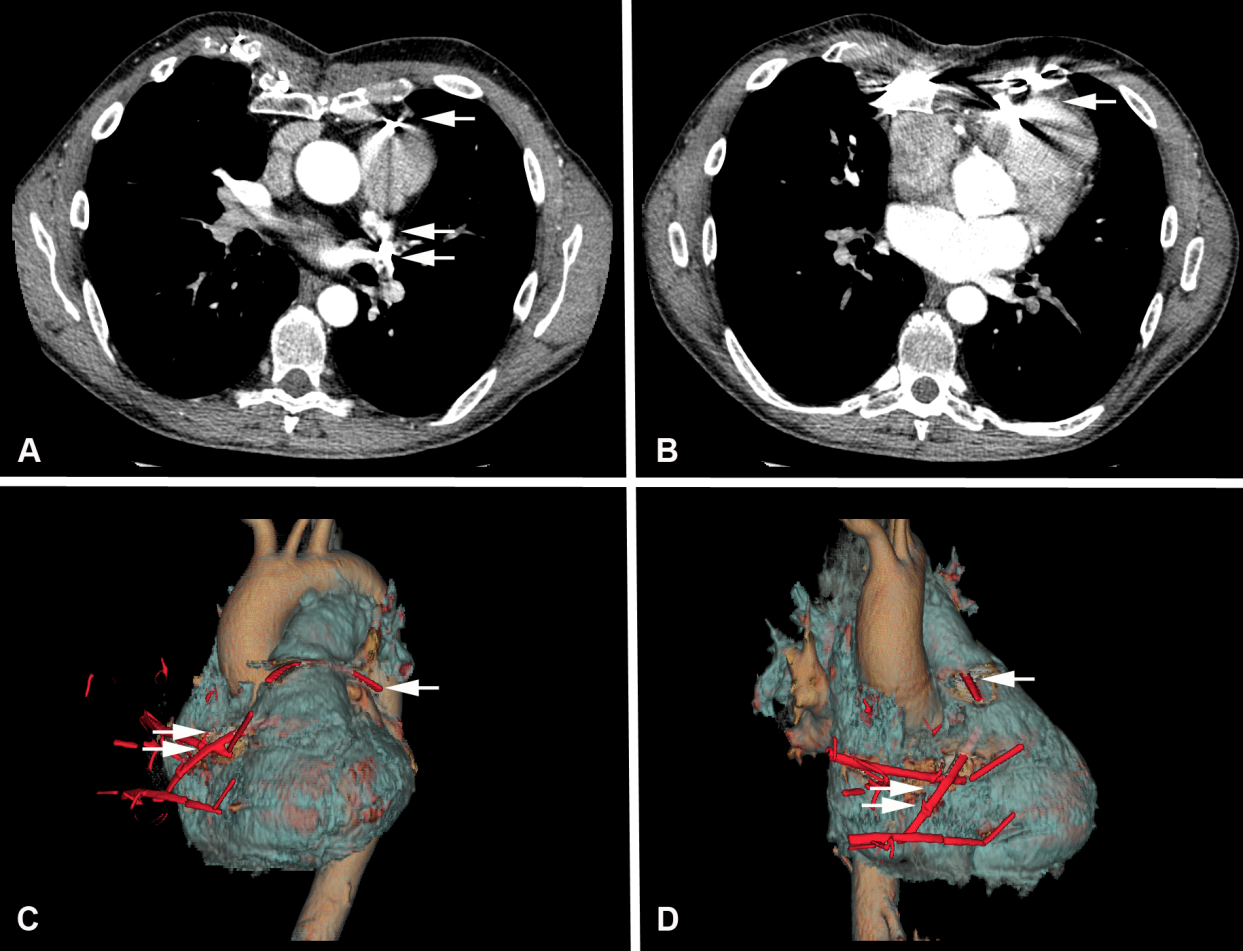
**The preoperative CT scan and 3-D reconstruction**. A: CT scan showed that the dorsal strut seemed to lie over the bifurcation of pulmonary trunk (single arrow). Its dorsal tip seemed to have perforated the left upper lobe bronchus (double arrow). B: The ventral strut lay behind the sternum. According to the traditional CT scan images, it was not apparent whether the struts perforated into the right ventricle or RVOT due to metal artifact. C and D: The following 3-D reconstruction of CT scan images revealed intracardiac migration of the two struts. The single arrow marked the dorsal strut, which perforated RVOT. The double arrow marked the ventral strut, which perforated into the right ventricle.

Preoperatively, the chest radiograph demonstrated multiple dislodged steel struts and wires. In the lateral view, several struts seemed to be dislocated in the middle mediastinum (Figure 1). CT scan showed the dorsal strut lying over the bifurcation of pulmonary trunk (Figure 2A). Its dorsal tip seemed to have perforated the left upper lobe bronchus. The ventral strut was displaced behind the sternum (Figure 2B). However, migration of struts into the right ventricle or RVOT was not readily discernable through traditional CT scan images due to metal artifacts. A 3-D CT scan image reconstruction revealed perforation of two struts into the right ventricle and RVOT (Figure 2C and 2D). An additional transthoracic echocardiography showed good left and right ventricular function without significant wall motion disorder. However, a significant pulmonary valve failure was noted.

**Figure 3:**
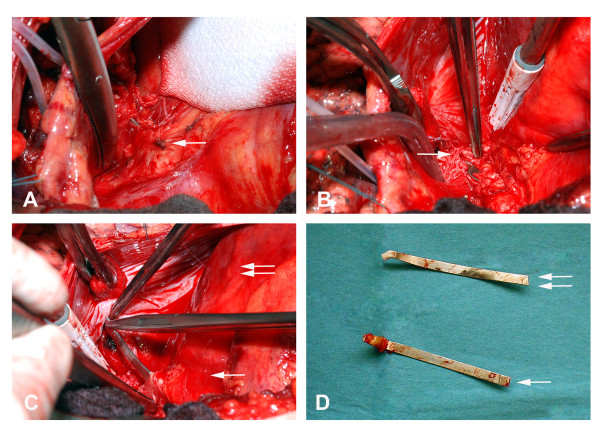
**Intraoperative photos**. A: After left antero-lateral thoracotomy and pericardiotomy a steel component was exposed right next to the pulmonary trunk marked by the single arrow. It was the dorsal strut perforating through the RVOT. B: The dorsal end of the strut was embedded in the wall of the left upper lobe bronchus (single arrow). C: The dorsal strut was pulled out followed by repair with running suture. The single arrow marked the pulmonary trunk and the double arrow marked RVOT. D: The photo showed the removed dorsal (single arrow) and ventral strut (double arrow).

Removal of the steel struts was undertaken under general anesthesia with endobrochial double lumen tube. After a left antero-lateral thoracotomy, pericardiotomy was performed over the left hilum with preservation of phrenical nerve. Careful dissection exposed a steel component right next to the pulmonary trunk (Figure 3A). Obviously, this was the dorsal strut perforating through the RVOT and pulmonary trunk. Its dorsal tip was embedded in the wall of the left upper lobe bronchus (Figure 3B). This finding was also confirmed by intraoperative bronchoscopy. Mobilization of the strut was performed by careful dissection of the embedded tip from the bronchial wall. The bronchial perforation was surgically repaired using single suture technique. Afterwards, the steel strut was cautiously extracted from pulmonary trunk. Under steady state of cardiopulmonary bypass (CPB), the strut was then pulled out, followed by repair with running suture (Figure 3C). Subsequently, the ventral steel strut was identified inside the right ventricle through palpation. Thereupon, CPB was established with cannulation of the left femoral vessels. After initiating ventriclular fibrillation, longitudinal right ventriculotomy was performed at RVOT followed by removal of the ventral strut. Ventriculotomy was then closed using single non-absorbable sutures in two layers. The patient was weaned off CPB with moderate inotropic support. After respiratory and hemodynamic stabilization, the patient was transferred from ICU to intermediate care unit on the fourth postoperative day. The further course was uneventful And the patient was discharged home on postoperative day 13.

## Discussion

As internal stabilization, metal struts or wires have been widely used in open PE repair. The struts are used to stabilize the sternum at the desired level, reduce the incidence of recurrent sternal depression and prevent paradoxical respiration [2]. Strut removal is recommended 6 to 12 months after repair [1-8]. Beside migration into pleural and peritoneal cavity, intrapericardial migration of dislodged metal struts or wires has been reported as an uncommon, but life-threatening sequela.

Reviewing the literature, in addition to the summary of Tahmassebi et al., only two case reports on intrapericardial migration of sternal wires or struts following open PE repair are presented so far [4]. Cope et al. reported a case of cardiac tamponade in a patient with sternal wire disruption after PE repair two years previously [5]. Unfortunately, the initial PE repair and surgical removal of the disrupted wire were not explicitly described. Therefore, this case report was not included in the present literature review (Table 1).

**Table 1:** Summary of the reported cases on intrapericardial migration of sternal wires or struts following open PE repair.

Author	Age (yrs)	Repair procedure	Migrated metal material	Injured structurs	Time of complication after repair (yrs)	Presenting symptoms	Surgical treatment	Approach	CPB	Outcome
Elami et al. 1991^8^	12	Liebermann procedure	Struts	Right atrium	2	Sudden chest pain and dyspnoea	Removal of parts of the metal plate and suture of the right atrium	Sternotomy	Steady-state	Survived

Dalrymple-Hay et al. 1997^1^	19	Ravitch	Struts	Right and left ventricular cavities	3/4	Progressive lower limb ischemia	Removal of the struts via aortotomy and atriotomy	Sternotomy	Yes	Survived

Onursal et al. 1999^3^	18	Ravitch	Struts	Right ventricle	4	Stabbing chest pain	Removal of the steel struts followed by pledgeted sutures	Right anterior thoracotomy	Steady-state	Survived

Barakat et al. 2004^7^	24	Morgan procedure	Wires	Epicardium	2	Cardiac tamponade	Removal of some wires	Sternotomy	Not mentioned	Survived

Mieno et al. 2010^6^	34	"sternal turnover"	Wires	Ascending aorta	28	Sudden chest pain	Resection and replacement of ascending aorta	Sternotomy	Yes	Survived

Present case report	53	Ravitch	Struts	Right ventricle, RVOT and left upper lobe bronchus	37	Progressive dyspnoea and malaise	Removal of the struts via ventriculotomy	Left anterior thoracotomy	Yes	Survived

RVOT = Right ventricular outflow tract; CPB = Cardiopulmonary bypass.

The previously reported intrapericardial migration of sternal struts or wires occurred between nine months and 28 years after initial open PE repair. In the present case, intracardiac migration of steel struts was found even 37 years after initial surgery. It underscores again the importance for early removal of sternal struts or wires, if chest wall stability is sustained. Distinguished from sternal wires, steel struts seemed to be able to migrate deeper and result in perforation into the cardiac cavities, probably due to their size and inflexible character. The presenting symptoms of intrapericardial injury are variable and could be unspecific. However, in three out of six cases, patients were admitted due to sudden chest pain. The intrapericardial injury was mostly described at the right ventricle. Dalrymple-Hay et al. reported a patient with multiple embolic events nine months after Ravitch repair[1]. The used steel strut migrated through the pericardium into the right ventricle, across the interventricular septum into the left ventricular cavity. Comparatively, migrations of metal material into the right ventricle were associated with less severe symptoms and could remain undiscovered for a long time, as described in the presented case.

Surgical approaches for removal of migrated materials in pericardium depend on the location of the struts and the injured structures. CPB support or steady state was required in most reported cases and the present case as well. The migrated material could be removed through directional pull followed by defect closure via running sutures. However, aortotomy and atriotomy were required for removal of the thrombus in the left ventricular cavity in the case reported by Dalrymple-Hay et al. [1] In the present case, ventriculotomy at RVOT was necessary for removal of the second strut. This emphasizes again the necessity to treat such life-threatening sequelae in a center of cardiothoracic surgery.

## Conclusion

In summary, intrapericardial migration of sternal struts or wires is a rare, but severe complication of open PE repair. Accurate localization of migrated materials by means of 3-D reconstruction of CT scan images and steady state of CPB are crucial for a successful surgical removal.

## Consent

Written informed consent was obtained from the patient for publication of this case report and accompanying images. A copy of the written consent is available for review by the Editor-in-Chief of this journal.

## Competing interests

The authors declare that they have no competing interests.

## Authors' contributions

RZ conceived the study, provided the information of the patient, performed literature search, wrote and reviewed the manuscript, CH was the operating surgeon of the patient and participated in the coordination of this study, DB participated in literature search, drafted the manuscript, TB operated the patient and participated in drafting the manuscript, JDS participated in literature search, drafted and reviewed the manuscript, AH participated in drafting the manuscript, supervised and reviewed the manuscript, MK participated in drafting the manuscript, supervised and reviewed the manuscript.

All authors read and approved the final manuscript.
